# The SNF2-Family Member Fun30 Promotes Gene Silencing in Heterochromatic Loci

**DOI:** 10.1371/journal.pone.0008111

**Published:** 2009-12-01

**Authors:** Ana Neves-Costa, W. Ryan Will, Anna T. Vetter, J. Ross Miller, Patrick Varga-Weisz

**Affiliations:** Chromatin and Gene Expression, Babraham Institute, Cambridge, United Kingdom; Oregon State University, United States of America

## Abstract

Chromatin regulates many key processes in the nucleus by controlling access to the underlying DNA. SNF2-like factors are ATP-driven enzymes that play key roles in the dynamics of chromatin by remodelling nucleosomes and other nucleoprotein complexes. Even simple eukaryotes such as yeast contain members of several subfamilies of SNF2-like factors. The FUN30/ETL1 subfamily of SNF2 remodellers is conserved from yeasts to humans, but is poorly characterized. We show that the deletion of *FUN30* leads to sensitivity to the topoisomerase I poison camptothecin and to severe cell cycle progression defects when the Orc5 subunit is mutated. We demonstrate a role of *FUN30* in promoting silencing in the heterochromatin-like mating type locus *HMR*, telomeres and the rDNA repeats. Chromatin immunoprecipitation experiments demonstrate that Fun30 binds at the boundary element of the silent *HMR* and within the silent *HMR*. Mapping of nucleosomes *in vivo* using micrococcal nuclease demonstrates that deletion of *FUN30* leads to changes of the chromatin structure at the boundary element. A point mutation in the ATP-binding site abrogates the silencing function of Fun30 as well as its toxicity upon overexpression, indicating that the ATPase activity is essential for these roles of Fun30. We identify by amino acid sequence analysis a putative CUE motif as a feature of FUN30/ETL1 factors and show that this motif assists Fun30 activity. Our work suggests that Fun30 is directly involved in silencing by regulating the chromatin structure within or around silent loci.

## Introduction

Chromatin is a complex superstructure that allows DNA fibres to be organized in the eukaryotic nucleus. Chromatin not only compacts DNA but also regulates its accessibility by allowing some sequences to be more available than others, e.g., to transcription factors [1]. The basic subunits of chromatin are the nucleosomes, 147 bp of DNA wrapped around eight histone proteins. Alterations of protein-DNA interactions in the nucleosome can occur by nucleosome remodelling catalyzed by specialized ATP-dependent enzymes [2]. Nucleosome remodellers alter chromatin structure by shifting nucleosomes from one position to another along the DNA (nucleosome sliding) [2]. In other cases they cause the disruption of nucleosomes or promote the assembly of new nucleosomes [2]. Some nucleosome remodelling factors also catalyze the exchange of histones within the nucleosome, for example, from a canonical histone to a histone variant [2]. A conserved ATPase domain is the defining feature of the SNF2 family of remodellers, named after its founding member, the budding yeast Snf2 transcriptional regulator. The SNF2 family is divided into subfamilies according to sequence similarities [3]. These include the well-characterized SWI2/SNF2, ISWI, CHD/Mi-2, SWR1 and INO80 subfamilies that all have been shown to target the nucleosome. The precise roles and activities of several of the remaining subfamilies are yet to be explored.

Here we characterize Fun30 from the budding yeast *Saccharomyces cerevisiae* and demonstrate that Fun30 promotes gene silencing in the silent HMR, telomeres and rDNA repeats. We provide evidence in support of a direct role of Fun30 in promoting silencing and show that the ATPase function of Fun30 is essential for its activity.

## Results

### Identification of a Novel Sequence Feature of Members of the FUN30/ETL1 SNF2-Subfamily

**Figure 1:**
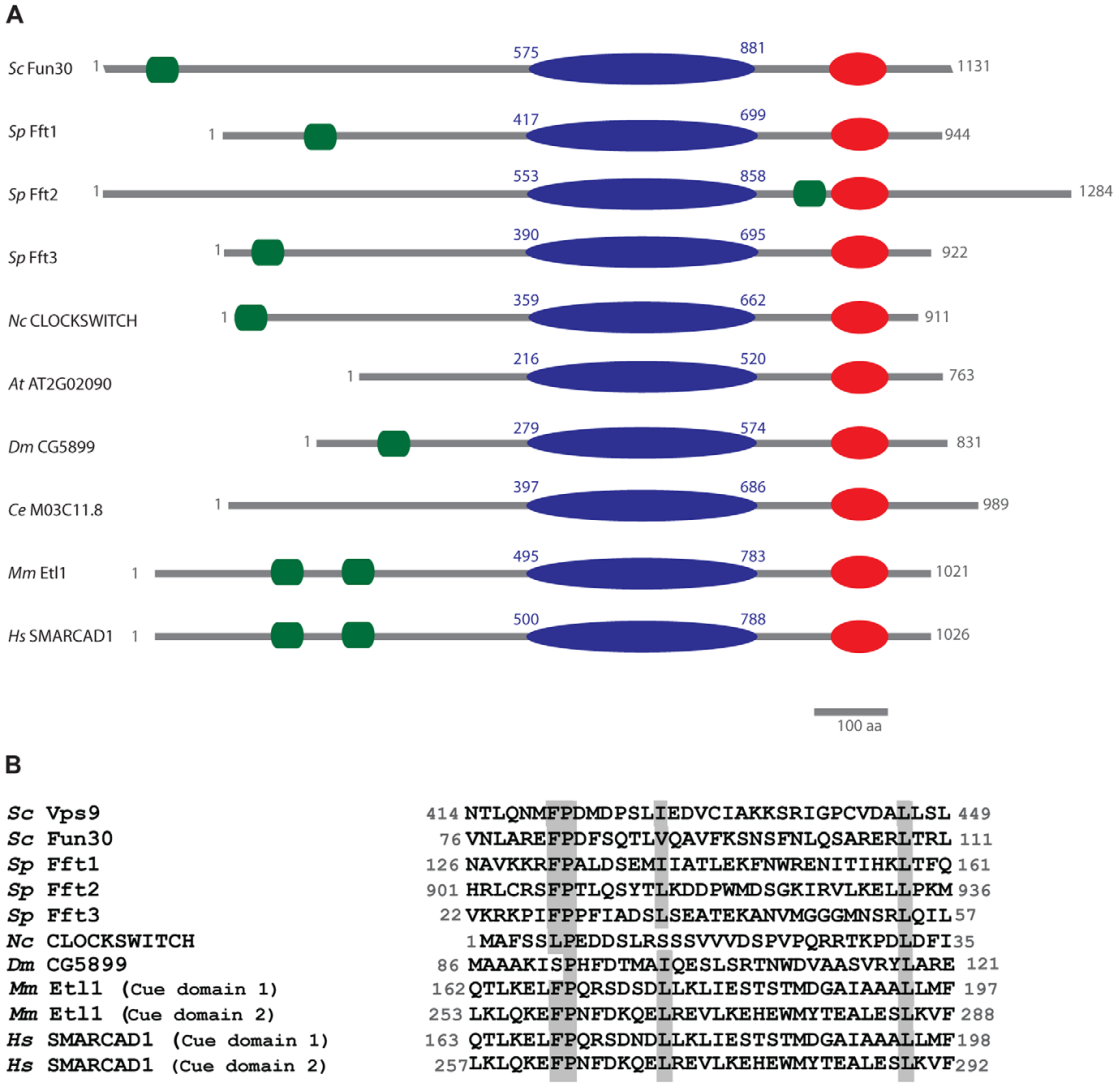
Fun30, Fft1-3, and SMARCAD1/ETL1 are SNF2-factors characterized by putative CUE motifs. A. Diagrams of *Saccharomyces cerevisiae* (*Sc*) Fun30, *Schizosaccharomyces pombe* (*Sp*) Fft1, Fft2, Fft3, *Neurospora crassa (Nc)* CLOCKSWITCH, *Arabidopsis thaliana (At)* AT2G02090, *Drosophila melanogaster (Dm)* CG5899, *Caenorhabditis elegans (Ce)* M03C11.8 and Human / mouse (*Hs, Mm*) SMARCAD1/ETL1 with relative positions of putative CUE motifs (green), SF2 ATPase (blue) and helicase-c domains (red). The amino acid positions for the SNF2_N domains are indicated. CUE motifs were not apparent in AT2G02090 and M03C11.8. B. Sequence alignments of the putative CUE motifs of Vps9, Fun30, Fft1-3, CLOCKSWITCH, CG5899, ETL1 and SMARCAD1. Conserved FP/LP and L positions are indicated by grey boxes.

Proteins of the SNF2 family are defined by the presence of specific conserved domains in addition to the ATPase domain, such as the bromodomain for the SWI2/SNF2 subfamily or the SANT domain for the ISWI subfamily [2]. A bioinformatics analysis of SNF2 subfamilies identified the FUN30 subfamily based on conserved features within the common helicase-like region [3]. We compared the sequences of SNF2 factors from budding and fission yeast by bioinformatic analysis, with focus on the regions outside the ATPase domains. This analysis also identified Fun30 from budding yeast as a member of a distinct subfamily that includes Fft (Fission yeast Fun Thirty) 1, 2 and 3 in fission yeast, mouse ETL1 and human SMARCAD1 [4], [5], the *Neurospora crassa* factor Crf10-1 (also called CLOCKSWITCH) [6], [7], the *Arabidopsis thaliana* AT2G02090, the *Caenorhabditis elegans* M03C11.8 and the *Drosophila melanogaster* G5899. To gain more information about defining protein sequence features of the FUN30 family, we compared Fun30 with the closely related Fft factors Fft1, Fft2 and Fft3 and the more distantly related budding yeast Snf2. The ATPase domain of Fun30 is very similar to the ATPase domains of the Fft factors in its sequence, and slightly less to Snf2 ATPase domain (Table 1). Similarly, the N- and C-termini of Fun30 exhibit a high degree of similarity to those of the Fft proteins, but less so to those of Snf2 (Table 1). The similarity between the C-terminal domains of Fun30 and the Fft factors is due to a predicted Helicase-c domain (Helicase conserved C-terminal domain), found in all SNF2 factors and in many other helicase-like proteins (Figure 1A). This domain may not necessarily mediate DNA unwinding, but may be involved in tracking along the DNA [8]. In the N-terminal domain of Fun30 we identified a putative UBA (Ubiquitin Associated domain)-like motif: the CUE motif (Coupling of Ubiquitin conjugation to ER degradation (Figure 1A). This motif, known to interact with ubiquitin (reviewed in [9]), stretches over 35 amino acid residues and is characterized by a phenylalanine (or leucine)-proline (FP/LP) separated by a defined number of amino acids from a leucine (L) [10]. The conserved regions have been shown to be required for ubiquitin binding and cellular functions, as observed for the prototypical CUE motif-containing protein in budding yeast, Vps9, involved in the endocytic pathway [11], [12], [13], [14]. The FUN30/ETL1 CUE motifs vary in their sequence: for example, the characteristic FP motif is in some cases replaced by the similarly hydrophobic LP motif, as described for other CUE motif-containing proteins (Figure 1B) [10].

**Table 1:** Identity between Fun30 protein sequence and the sequences of the related Fft proteins and of Snf2.

	N- and C-ter	ATPase
Fun30	100^*^	100^*^
Fft1	62.1	51.1
Fft2	45.5	56.6
Fft3	63.9	48.1
Snf2	22.5	37.9

Each protein sequence was compared with Fun30 sequence and the results are presented in percentage identity. The complete sequences of the proteins were divided in shorter sequences and analysed separately: N-ter refers to the sequence from the beginning of the protein until the beginning of the ATPase domain; ATPase refers to the entire ATPase domain; C-ter refers to the sequence from the end of the ATPase domain until the end of the protein. The results for N-ter and C-ter were added and are shown in N- and C-ter column.

We investigated if the CUE domain is a unifying feature of the FUN30/ETL1 subfamily. Fft1 and Fft3 have a putative CUE motif in their N-terminal regions, similar to Fun30. In contrast, a putative CUE motif in Fft2 is located downstream of the ATPase domain. In the homologous mouse ETL1 and human SMARCAD1 factors we identified putative tandem CUE motifs N-terminal to the ATPase domain (CUE motif 1 and CUE motif 2, Figure 1A, B). The CUE motif 1 and CUE motif 2 are identical between mouse and human (Figure 1B) and separated by around 50 amino acid residues that differ in sequence from mouse to human (not shown). It is possible that the two CUE motifs in SMARCAD1/ETL1 function cooperatively, as CUE motifs have been shown to function as dimeric modules in other proteins [13], [15]. We could not identify potential CUE motifs in the *Arabidopsis thaliana* AT2G02090 and the *Caenorhabditis elegans* M03C11.8. In conclusion, we found that the putative CUE motif is conserved in several FUN30/ETL1 factors ranging from yeast to human.

### Deletion of *FUN30* Renders Cells Sensitive to Topoisomerase I Inhibitor Camptothecin

**Figure 2:**
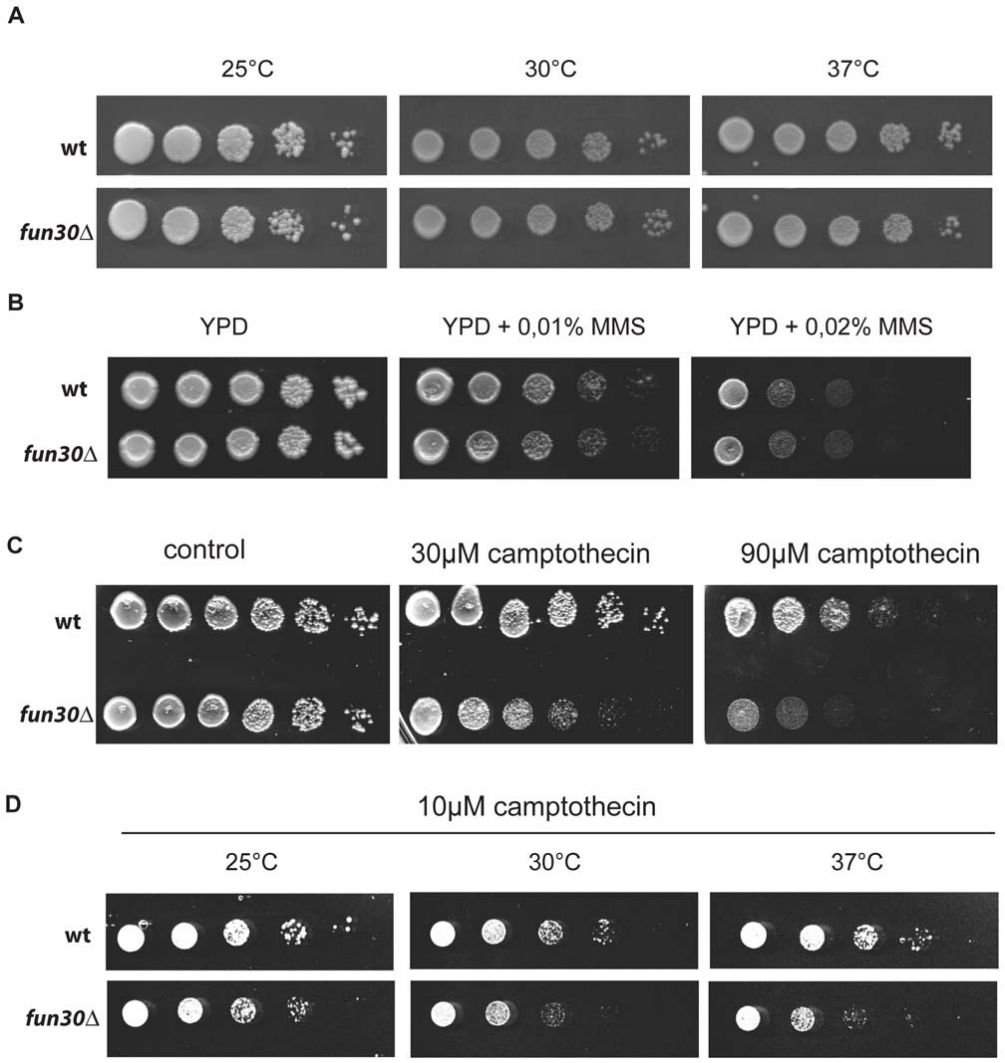
Deletion of *FUN30* affects viability on camptothecin containing media. A. Strain Y00000 (wt) and Y00389 (*fun30*Δ) were spotted after ten-fold serial dilution on rich medium agar (YPD) and grown for 2 days at the indicated temperatures. B. Same as in (A) but plates contained the indicated amounts of methylmethanosulfonate (MMS) and were incubated for 3 days at 30°C. C. Same as in (A) but serial dilution was five-fold on YPD, 1% DMSO (control) or YPD, 1% DMSO and indicated amounts of camptothecin. Growth was at 30°C. D. Same as in (A) but plates contained 10 µM camptothecin and were incubated 3 days at indicated temperatures.

Deletion of *FUN30* does not affect cell viability in normal conditions ([16], Figure 2A). We tested if deletion of *FUN30* affected cell growth under various stress conditions, including environmental stress (elevated temperature, heatshock, formamide, osmotic stress, hydrogen peroxide) and nutritional stress (carbon-, nitrogen-, sulphate-, inositide- starvation). We did not find that deletion of *FUN30* affected viability in these assays, as shown for growth under elevated temperature (Figure 2A, data not shown). This is in contrast to a previous study that indicated temperature sensitivity of *fun30*-deleted cells [16]. The discrepancy may be explained by different strain backgrounds used in this study. Thiabendazole which causes destabilization of mitotic spindles did not have a significant effect on *fun30*-deleted cells (not shown). Two high-throughput screens indicate genetic interactions between *FUN30* and genes involved in protein transport between the Golgi apparatus and the endoplasmatic reticulum, ER [17], [18]. To further examine this finding, we grew wildtype and *fun30*-deleted cells in the presence of brefeldin A, a drug that interferes with ER-Golgi transport. However, we did not observe a significant difference in viability (not shown). Mutant cells that are defective in transcription elongation often show enhanced sensitivity to 6-azauracil, but *fun30*-deleted cells did not show this (not shown). The deletion of *FUN30* has been reported to lead to a modest increase in survival after UV irradiation, suggesting a role in the DNA damage response [19]. However, we did not observe growth differences between control cells and *fun30*-deleted cells under several DNA damage-inducing conditions tested, including growth in the presence of methylmethanosulfonate (MMS, Figure 2B), etoposide, amsacrine, zeocin, and hydroxyurea. Interestingly, deletion of *FUN30* caused a mild, but reproducible growth defect in the presence of topoisomerase I inhibitor camptothecin (CPT), especially when grown at an elevated temperature (Figure 2C, D). CPT binds topoisomerase I, trapping it as a reaction intermediate covalently bound to DNA. This is thought to form ‘roadblocks’ for the transcription and DNA replication machineries tracking along the DNA, leading to DNA double strand breaks if not resolved.

### *FUN30* Interacts Genetically with ORC to Promote Cell Cycle Progression

**Figure 3:**
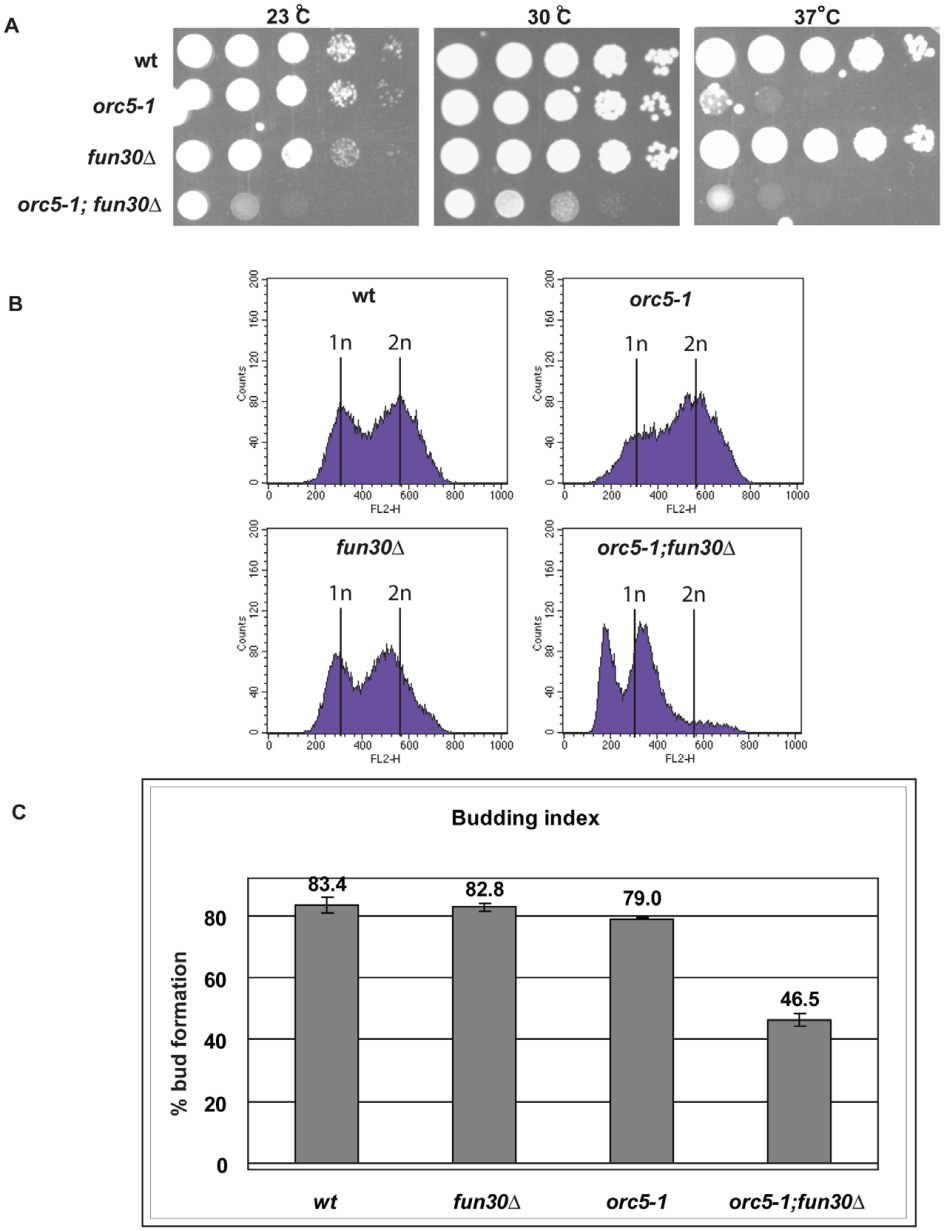
*FUN30* interacts genetically with ORC in cell cycle progression. A. *FUN30* was deleted in strains JRY7385 (wt) and JRY7459 (*orc5-1*)[20]. The deletion and source strains were spotted onto rich medium (YPD) agar plates after a 10-fold serial dilution series and incubated for 2 days at the indicated temperatures. B. Flow cytometry profiles, measuring DNA content versus cell number, were determined for the strains described above. 1n and 2n indicates the cellular DNA content, unreplicated and replicated, respectively. C. The budding index of the strains described in A was determined as the percentage of cells with buds in an exponentially growing cell population in rich medium at 30°C. The results are the average of three independent cultures for each strain and standard deviation.

A previous study used a synthetic lethality screen to discover genes that genetically interact with temperature sensitive mutations in the ORC2 and ORC5 subunits. This screen identified *FUN30* among several other genes [20]. Because this interaction was not further characterized, we deleted *FUN30* in the *orc5-1* mutant background. We observed that the double mutant cells exhibited poor growth at 23°C, 30°C, and 37°C, whereas *orc5-1* single mutants show normal growth at 23°C and 30°C, but not at 37°C and the *fun30* mutant cells did not show any remarkable temperature sensitivity (Figures 2 A, 3 A). Cell cycle analysis by flow cytometry showed that *fun30*-deletion mutants do not exhibit a noticeable defect in cell cycle progression (Figure 3 B, *w*t, *fun3*0*Δ*). This was also confirmed using synchronized cells (data not shown). The *orc5-1* mutants exhibited some alteration in the cell cycle profile as previously reported [21] (Figure 3 B, *orc5-1*). However, the *orc5-1, fun30Δ* double mutant showed what appears to be drastic defects in their cell cycle profile, with cells apparently accumulating in G1-early S phase with under-replicated DNA (genome content between 1n and 2n) and a substantial number of cells showing a genome content below 1n (Figure 3 B, *orc5-1, fun30Δ*). However, it is not uncommon that yeast DNA FACS profiles display artifactual shifts to what appears lower DNA content. Therefore, this could conceivably mean that the *orc5-1; fun30Δ* double mutants' FACS profile is the same as wildtype, indicating that the *FUN30* deletion is epistatic to the *orc5-1* G2 phenotype. To resolve this issue, we scored bud formation, which is tightly linked to cell cycle progression in budding yeast and can be used to monitor defects in the cell cycle. Scoring the number of cells with a bud showed that the *orc5-1, fun30Δ* double mutants exhibit an almost 2 fold reduction of cells with a bud, indicating a major defect in cell cycle progression, most likely because cells do not enter S phase (Figure 3 C). In summary, these findings indicate that ORC and *FUN30* interact genetically to promote cell cycle progression.

### Fun30 Is Involved in Silencing at a Heterochromatic Locus, *HMR*

**Figure 4:**
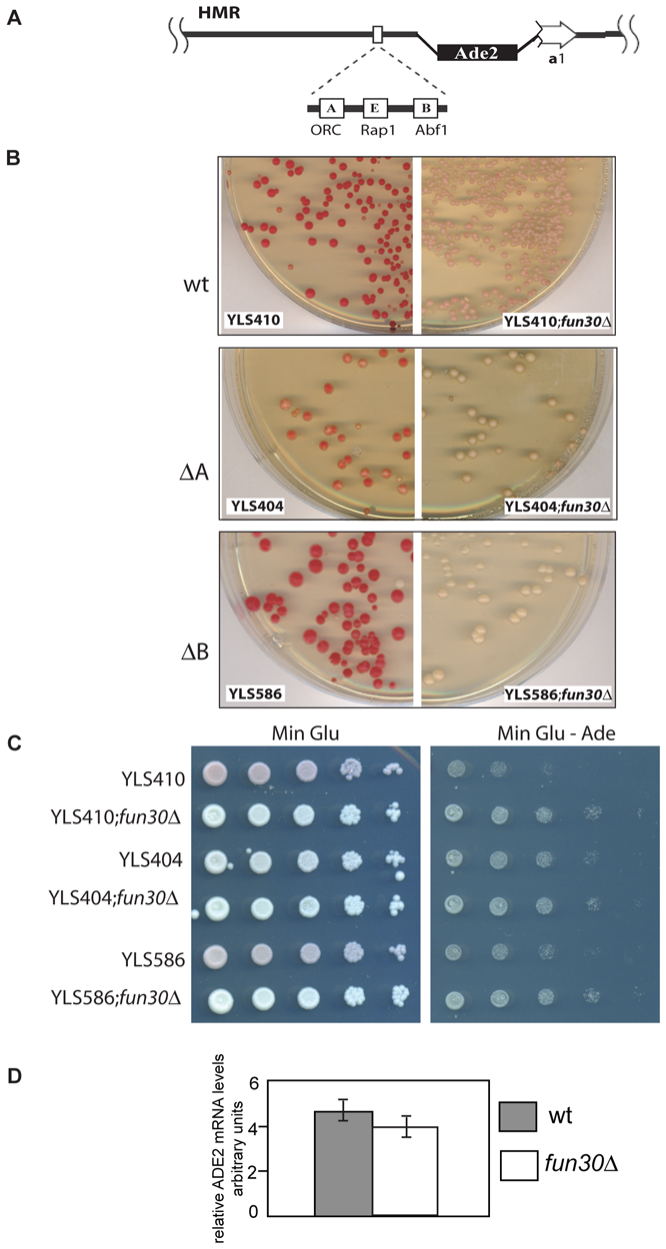
Fun30 is involved in silencing in the *HMR* locus. A. Diagram of the *HMR* silencing reporter (adapted from [32]). B. Colonies of the strains YLS410 (wildtype silencer, [32]), YLS404 (ΔA silencer, [32]) and YLS586 (ΔB silencer, [32]) turn red because of lack of expression of *ADE2*. When *FUN30* is deleted in these backgrounds, the colonies stay white, indicating derepression of *ADE2*. C. The role of Fun30 in repressing the *ADE2* reporter embedded in the *HMR* is tested by monitoring growth in the absence of adenine. The repression of the *ADE2* reporter gene in the ‘wt’ strain (YLS410) leads to reduced growth compared to the *fun30Δ* isogenic strain. 10-fold serial dilutions of cells were spotted on agar plates with minimal glucose medium (MinGlu) or minimal glucose medium without adenine (MinGlu-Ade) and incubated for 3 days at 30°C. D. Fun30 does not regulate *ADE2* in its endogeneous locus. The amount of *ADE2* mRNA expressed from its endogenous locus in control (JS311, [37]) and its *fun30*-deleted sister strain was determined after reverse transcription of the mRNA and quantitative PCR and normalized to *ACT1* expression. Shown is the average of two experiments with standard deviation.

Several genes identified in the ORC synthetic lethality screen have been linked to gene silencing, including *ASF1*, *HST1*, *HST3*, and *SUM1* [20], [22], [23], [24], [25], [26], [27], [28], [29], [30]. Therefore, we tested if Fun30 is involved in gene silencing at the silent mating-type locus *HMR*. This is a well characterized locus where heterochromatin-like structures are assembled in budding yeast [31]. Repression of the *HMR* locus requires several *trans*-acting factors and *cis*-acting sequences called silencers that flank this region (reviewed in [31]). The *HMR-E* silencer is necessary and sufficient for repression and consists of three sites (A, E, and B), which are partially redundant and bind ORC, Rap1p and Abf1p (reviewed in [31]). We used a strain with an *ADE2* reporter gene insertion in the silent mating type locus *HMR*, with the endogeneous *ADE2* mutated [32]. Silencing of the *ADE2* gene results in red colonies, whereas a loss of silencing results in white colonies [32]. Silencing was also examined in strains containing mutations in either the ORC binding site or the ABF1 binding site of the *HMR* silencer (Figure 4A, [32]). Deletion of *FUN30* resulted in a loss of silencing of *ADE2* in the *HMR*, whereas the cells expressing Fun30 maintained silencing when the silencer element was intact (Figure 4B). The degree of silencing appeared to decrease as silencer elements were mutated, as indicated by pink rather than red colonies, but deletion of *FUN30* de-repressed *ADE2* even further giving rise to white colonies (Figure 4B). A similar assay that monitors viability in the absence of adenine confirms that Fun30 is involved in silencing, because the *fun30* mutant cells grow better in the absence of adenine (Figure 4C). However, in this assay the deletion of the ORC-binding site, the deletion of *FUN30*, or the double mutation result in almost the same growth, indicating possible epistatic relationship between ORC and Fun30 at this locus. RT-PCR analysis indicates that Fun30 does not regulate *ADE2* when it is at its endogenous locus (Figure 4D), showing that its silencing function is linked to the repressed state of the *HMR* locus.

### Fun30 Is Involved in Silencing of Reporter Genes at Telomeres and within the rDNA Repeats

**Figure 5:**
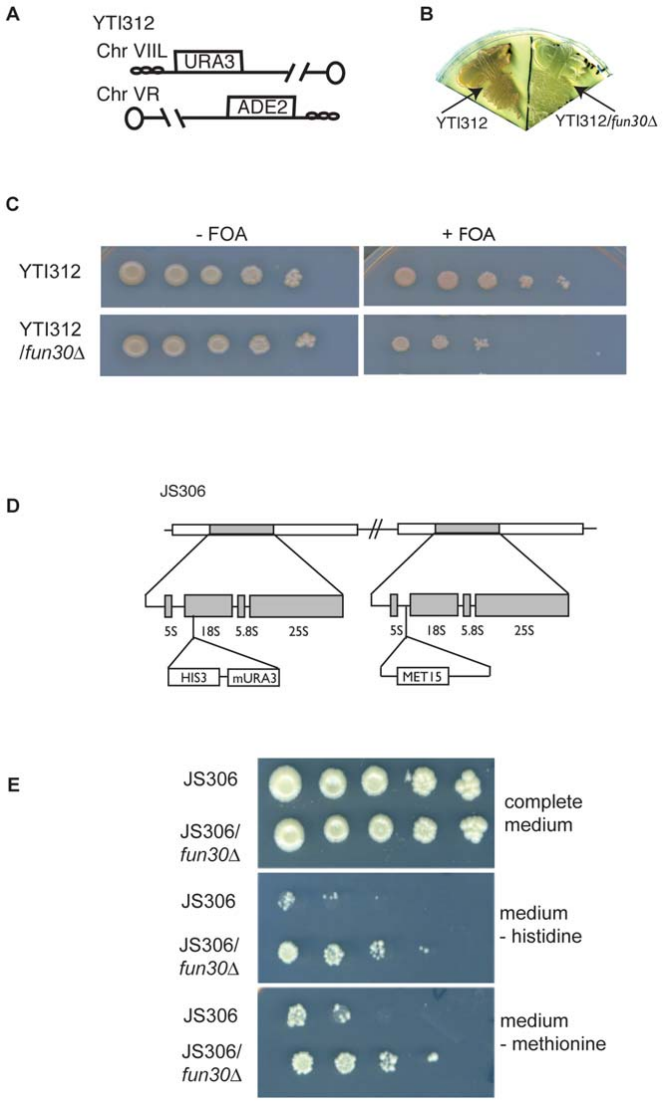
Fun30 is involved in silencing at telomeres and the rDNA repeats. A. Diagram of the telomeric reporter genes in strain YTI312 (adapted from [35] oval: centromere, open boxes:*URA3* and *ADE2* reporter genes, small ovals: telomere. B. Silencing of the telomeric *ADE2* (YTI312, [35]) in the presence but not in the absence of Fun30 (YTI312;*fun30*Δ). C. Analysis of role of Fun30 in telomeric silencing of a *URA3* reporter by serial dilution assay on plates without or with 5-FOA. D. Representation of the rDNA structure of strain JS306 (adapted from [37]). E. Analysis of role of Fun30 in silencing of *HIS3* or *MET15* reporter genes within the rDNA locus by serial dilution assay on plates with complete synthetic media or media lacking either histidine or methionine.

We tested if Fun30 has a general role in promoting silencing within heterochromatin like regions in yeast, affecting regions other than the *HMR*, such as the telomeres and rDNA repeats [31], [33], [34]. Reporter assays in *fun30*-deleted cells containing an *ADE2* reporter gene inserted in telomeric region of the right arm of chromosome V and an *URA3* reporter gene inserted close to telomere of the left arm of chromosome VII while the endogeneous genes were inactivated ([35], Figure 5A) showed that *FUN30* is involved in telomeric silencing (Figure 5 B, C).

The budding yeast rDNA locus consists of a tandem array of 100-200 repeats of 9.1 kbp units on chromosome XII. Silencing of Pol II-transcribed reporter genes occurs within the rRNA locus, even though this region is actively transcribed by Pol I and Pol III [31], [34], [36]. We used reporter assays, where the genes *HIS3* (conferring auxotrophy in the absence of histidine added to the media), *URA3* (conferring auxotrophy in the absence of uracil or sensitivity to 5-Fluoroorotic acid, 5-FOA) and *MET15* (conferring auxotrophy in the absence of methionine) have been inserted within rDNA repeats (with the endogeneous genes inactivated), to test if Fun30 is involved in silencing there [37] (Figure 5 D). Deletion of *FUN30* significantly improved the growth of the cells in the absence of histidine or methionine, indicating the Fun30 is involved in rDNA silencing (Figure 5 E). The effect of deletion of *FUN30* on growth minus uracil or survival in the presence of 5-FOA was less pronounced (not shown). In summary, our data support a general role of Fun30 in promoting silencing in budding yeast.

### Fun30 Is Enriched at the *HMR* Heterochromatin and Boundary Element and Regulates Chromatin at the *HMR* Barrier Element

**Figure 6:**
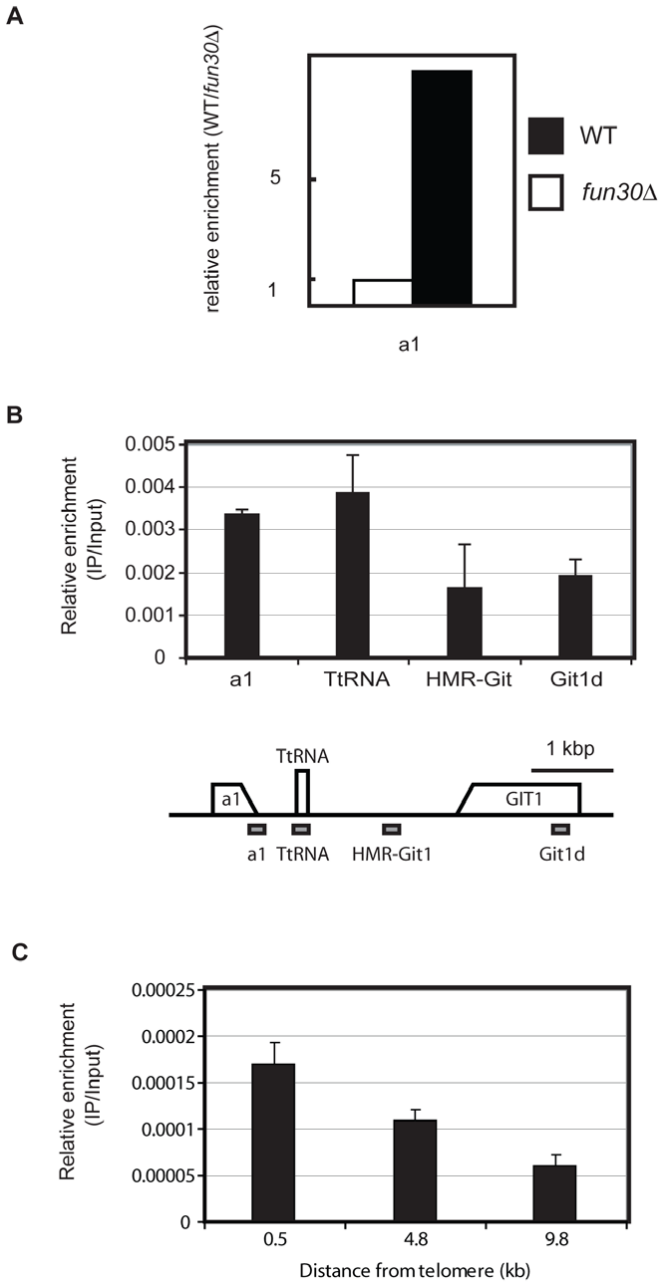
Fun30 binds at the silent *HMR* locus. A. Validation of the specificity of the affinity purified Fun30 antibody for chromatin immunoprecipitation (ChIP). Control (wt) and *fun30Δ* cells were analyzed by ChIP with Fun30 antibody. Immunoprecipitated DNA was quantified at the a1 locus using quantitative PCR, corrected for background signal using the IgG control, and normalized relative to input. Shown is a representative result of two repeat experiments. B. upper panel: ChIP analysis of Fun30 occupancy across the *HMR* barrier. Error bars are standard deviation of the two biological replicates of this experiment. Lower panel: Diagram of the *HMR* barrier region. Arrows indicate the orientation of specific genes in the region. Small bars underneath indicate the relative position of qPCR probes used in ChIP analyses. C. Fun30 occupancy near the telomere VIR.

As a next step we examined if Fun30 is directly involved in silencing by binding within or at silenced loci and mediating changes of chromatin structure there. We focused this analysis on the telomere-proximal side of *HMR*, because it contains a defined boundary element, a tRNA, which creates a sharp transition between heterochromatin and euchromatin [38]. We used chromatin immunoprecipitation to test if Fun30 binds to the silent *HMR*, including the boundary region and regions outside of the heterochromatin. Chromatin immunoprecipitation studies were performed using an affinity-purified polyclonal antibody generated against Fun30. This antibody is specific for Fun30, because it did not immunoprecipitate chromatin from a *fun30*-deletion strain (Figure 6A). Consistent with a direct role of Fun30 in determining heterochromatin structure, Fun30 occupancy was increased at the barrier tRNA gene, as well as further upstream at the silent a1 locus (Figure 6B). Fun30 occupancy was decreased in a region between the tRNA gene and the *GIT1* gene and within the coding region of *GIT1* (Figure 6B). This suggests that Fun30 directly associates with the heterochromatic *HMR* and the boundary element, influencing chromatin structure there. We tested Fun30 occupancy at telomere VIR and found that occupancy, as measured by immunoprecipitation efficiency compared to input, was overall significantly lower compared to the *HMR* locus. However, within that site, occupancy was highest close to the telomere end and progressively dropped at loci towards the centromere (Figure 6C).

**Figure 7:**
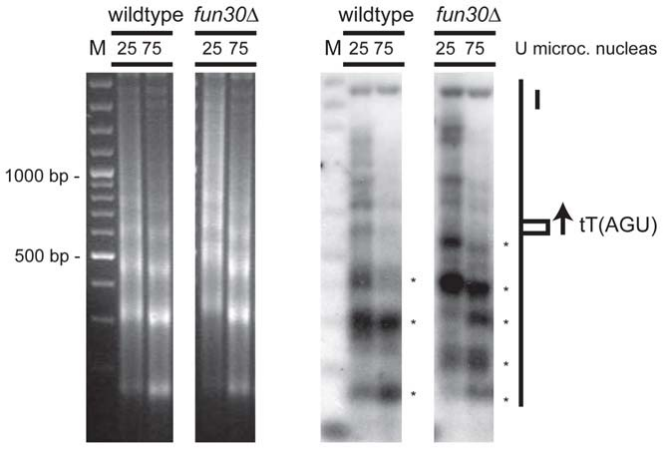
Fun30 regulates chromatin structure at the *HMR* boundary element Chromatin analysis using micrococcal nuclease digestion and indirect endlabeling. Spheroplasts were generated from both a wild-type yeast strain and the isogenic *fun30* mutant strain and incubated briefly in the presence of increasing concentrations of micrococcal nuclease. The purified genomic DNA was then separated on an agarose gel and analyzed after ethidium bromide staining (left panels) or by Southern blot analysis for the *HMR* boundary region via indirect end-labelling (right panels). Asterisks indicate major digestion products. The position of the tRNA barrier is indicated by a small box. The arrow indicates the direction of transcription of the tRNA. The coding regions for a1 and *GIT1* lie upstream and downstream, respectively, of the analysed fragment. The molecular size marker (M) is in multiples of 100 bp.

Micrococcal nuclease analysis was performed on chromatin of yeast nuclei to test if Fun30 affects chromatin structure at the *HMR* locus. Deletion of *FUN30* did not detectably alter global chromatin structure (Figure 7, left panels). Probing digested chromatin for the region flanking the *HMR* boundary revealed an increase in nuclease sensitivity at the tRNA gene and within the *HMR* in *fun30*-deleted strains compared to the isogenic wildtype strain, in line with the notion that Fun30 is required to create a restrictive chromatin structure (Figure 7, right panels).

**Figure 8:**
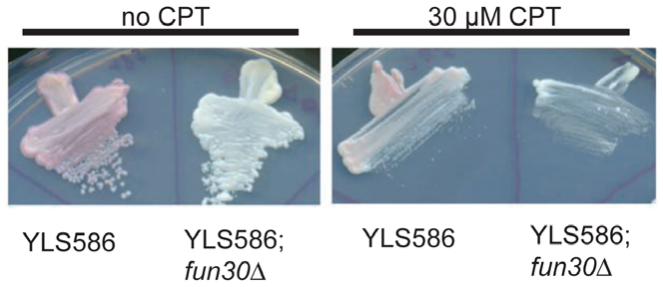
Camptothecin does not abrogate silencing within the *HMR*. Silencing reporter strains YLS586 or YLS586; *fun30*Δ (see Figure 4) were grown on minimal glucose medium, 1% DMS0 with or without 30 µM camptothecin (CPT) and the accumulation of red pigment, indicative of silencing of the *ADE2* reporter gene embedded in the HMR, was monitored as in Figure 4.

We used the *HMR* silencing assay in the presence of camptothecin to determine whether the *fun30* null allele has a direct effect on silencing or if the silencing phenotypes are caused indirectly by *fun30* through constitutive activation of a pathway(s) normally induced by the presence of roadblocks such as single strand nicks with a covalent DNA-protein bond. We found that camptothecin at levels that inhibited growth of *fun30*-deleted cells did not affect the *HMR* gene silencing in control cells as monitored by the *ADE2* reporter assay (Figure 8). In conclusion, our data provide evidence for a direct role of Fun30 in silencing at the *HMR*.

### ATP-Binding Site Is Essential and the CUE Motif Supportive for Activity of Fun30

**Figure 9:**
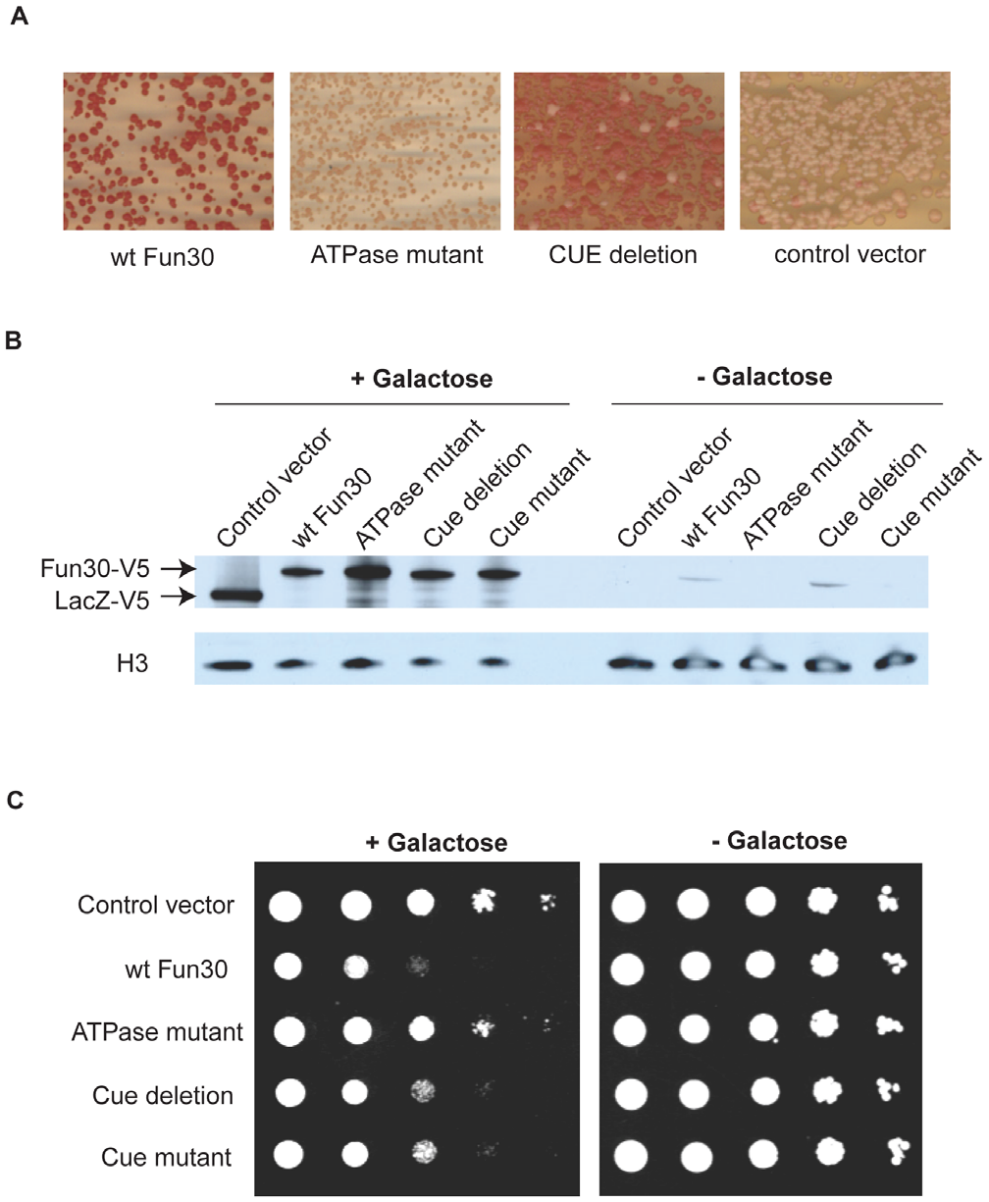
Role of ATPase function and putative CUE-motif in Fun30 activity. A. Silencing of the *ADE2* reporter in YLS586; *fun30Δ* is restored by expressing wildtype Fun30 *in trans*, but not by a Fun30 ATPase mutant or by a control vector expressing LacZ. Expression of Fun30 with the putative CUE motif deleted does not restore silencing as monitored by red pigment formation. Expression was in galactose at 30°C, driven by the galactose-inducible promoter of *GAL1*. B. Immunoblot analysis of the Fun30 protein *in trans*-expression with anti-V5 antibody. An immunoblot for histone H3 served as protein loading control. V5-tagged Fun30 and indicated mutants thereof were expressed *in trans* in a galactose- or glucose dependent manner in Fun30 containing strain JRY7385 from multicopy plasmids, the control vector expresses V5-tagged beta-galactosidase. C. Cells from (B) were serially diluted 10-fold, spotted on media with galactose or with glucose and grown for 3 days at 30°C.

The ATPase activity of Snf2-like factors is essential for most of their biological functions, and point mutation of critical residues involved in ATP binding usually results in the same phenotype as the full deletion of the gene [39], [40], [41]. Likewise, the deletion or mutation of critical domains flanking the ATPase, such as the bromodomain in SWI/SNF, has been shown to affect the function of the protein in many instances [42], [43], [44]. We tested the role of Fun30's ATP-binding site and putative CUE motif for silencing in the *HMR*. Expression *in trans* was from a multi-copy plasmid under the control of the inducible *GAL1* promoter. Complementation with the wildtype Fun30 in the *fun30*-deleted strain restored the silencing of the *ADE2* reporter embedded in the *HMR* (Figure 9A). Complementation with an ATPase binding site point mutant of the Walker A motif (GKT to GRT) or a negative control vector did not (Figure 9A). Deletion of the CUE motif in the expression construct (deletion of aminoacids 73 to 111) resulted in pink and white colonies, indicating that silencing was not as strong and stable with this mutant Fun30 (Figure 9A, CUE deletion). The experiments shown in Figure 9A were performed with cells grown on galactose for full induction of Fun30 expression. Expression levels between wildtype, ATPase mutant Fun30 and the CUE deletion mutant Fun30 were similar in this condition (Figure 9B). The low-level, uninduced expression of Fun30 also rescued silencing (not shown). Therefore, Fun30 is involved in silencing an RNA polymerase II dependent reporter gene when embedded in a heterochromatin-like locus, the ATPase activity of Fun30 is essential for its silencing activity and the CUE motif assists in full silencing.

Overexpression of Fun30 has been shown to result in poor cell viability, possibly because of chromosome instability [16]. We tested if the ATPase function and the putative CUE motif are involved in this phenotype by overexpressing wildtype and mutant Fun30 using the constructs described above (Figure 9B). When wildtype Fun30 expression was induced with galactose, cell growth was severely impaired when compared with cells expressing the control protein β-galactosidase (Figure 9C). The toxicity of the protein was dependent on the ATPase activity, as overexpression of the ATPase point mutant of Fun30 did not affect cell growth, suggesting that the point mutation in the ATPase mutant impairs the activity of the protein (Figure 9C). To test the importance of the CUE motif for the toxicity phenotype, we overexpressed the CUE-deletion mutant, as well as a version of Fun30 in which the residues that are critical for ubiquitin binding in other CUE motifs have been mutated (aminoacids 82 and 83: FP mutated to AA, the equivalent residues have been shown to bind ubiquitin in the CUE motifs of other proteins [12], [14]). The results show that both the overexpression of the CUE-deletion and the CUE-point mutant protein caused diminished growth (Figure 9C). However, the deleterious effect of over-expression of these mutants was less than that caused by the full-length protein.

## Discussion

We show that Fun30 is required for silencing of reporter genes embedded within transcriptionally repressed domains, the silent *HMR*, telomeres and rDNA repeats. The ATPase function appears essential for this activity, because mutating an essential lysine residue in the ATPase domain abrogates this function. A toxicity assay upon overexpression reinforces the importance of the ATPase for Fun30 activity. In budding yeast, ATP-dependent chromatin remodelling factors ISW1 and ISW2 have been linked to gene silencing within the *HMR* and rDNA, but not telomeres, whereas the SWI/SNF complex has been shown to be required for rDNA and telomeric silencing, but not silencing within the *HMR* and *HML* loci [30], [45], [46], [47]. Therefore, silencing appears to be the result of the concerted action of several remodelling factors. Fun30 appears to be special in that it affects silencing in the *HMR*, telomeres and rDNA loci. We can presently not exclude an indirect role of Fun30 in heterochromatin maintenance. However, we favour a direct role, because we find that Fun30 binds at the silent *HMR* locus, and less so to the adjacent euchromatic region. Furthermore, we find that deletion of Fun30 results in an altered chromatin structure at the *HMR* boundary element. Future work will establish if Fun30 promotes gene silencing in heterochromatin by assisting the establishment of specific histone modifications (e.g., histone hypoacetylation), by regulating histone variants, or heterochromatin-specific non-histone proteins.

How is the role of Fun30 in heterochromatin connected with its genetic interaction with ORC? Our data suggest that deletion of the ORC binding site of the silencer element does not completely abrogate a role of Fun30 in silencing within the *HMR*. This is because there is some difference in the accumulation of red pigment (indicative of silencing) between cells containing single deletions of either the ORC binding site or *FUN30*, or the double mutant in this sensitive assay (see Figure 4B, accumulation of red pigment is apparent even when growth is not compromised). However, an assay that monitors growth in the absence of adenine (and that maybe somewhat less sensitive) indicates at least some epistatic interaction between the role of Fun30 in *HMR* silencing and the ORC binding site within the silencer, because there is almost no difference in growth between single deletion strains at the ORC site or *FUN30*, or the double deletion strains (Figure 4C). Therefore, there may be an interaction between Fun30 and ORC at the heterochromatic regions, and this, in turn, may be important for the silencing-related function of Fun30 and the replication-related function of ORC at these sites: The ORC complex may be involved in recruiting Fun30 to establish a silent chromatin configuration within the *HMR* or Fun30 may facilitate the binding of ORC within the silencer element and other sites within the genome. If the number of functional ORC complexes is diminished, as in the *orc5-1* mutation, the supportive role of Fun30 for ORC establishment may become apparent. Alternatively, Fun30 may promote cell cycle progression in a parallel pathway to ORC function, e.g., by regulating histone metabolism or facilitating DNA polymerase progression through chromatin. However, it is noteworthy that the deletion of *FUN30* does not affect viability in the presence of hydroxyurea (data not shown), a drug that inhibits ribonucleotide reductase, leading to diminished deoxyribonucleotide pools and DNA replication stress. If Fun30 has a role in facilitating replication through chromatin, one would expect that the deletion of it would aggravate the deleterious effect of hydroxyurea, as is the case with Ino80, a chromatin remodelling factor implicated in facilitating chromatin replication [48], [49], [50]. It is interesting that when other genes involved in silencing are deleted, they cause synthetic lethality or sickness with ORC mutants [20]. Additional genes involved in such synthetic interactions with ORC mutants are involved in cohesion [20]. An interesting suggestion might be that silencing established by Fun30 and other factors such as ASF1 is linked to some aspect of cohesin function.

We attempted to obtain further insight into the biological function of Fun30 by identification of interaction partners using TAP (Tandem Affinity Purification) of endogenously tagged Fun30, but did not find any interaction partner using this approach (data not shown). This may indicate that Fun30 does not form a heteromeric complex as so many other chromatin remodelling factors [2]. Another explanation may be that such a complex is only formed under specific circumstances or is not extractable from the chromatin under our conditions.

Several members of the FUN30/ETL1 family of remodellers are characterized by the presence of putative CUE motifs. CUE motifs of other proteins bind to polyubiquitin chains, but also to monoubiquitin *in vitro* [12]. The binding to ubiquitin is required for the function of CUE motif-containing proteins, for example, in intracellular trafficking [11], [12]. Moreover, the CUE motif can mediate intramolecular monoubiquitination [9], [12], [14]. Here, we provide evidence that the putative CUE motif of the budding yeast Fun30 is required for the full function of the protein. In chromatin, regulation by ubiquitin assures genomic stability and cellular maintenance. Relevant examples of ubiquitinated proteins include histones [51], PCNA [52], RNA PolII [53], numerous transcription factors [54] and various components of the DNA repair machinery [55]. It will be interesting to determine if the function of Fun30 is dependent of the recognition of ubiquitinated factors, e.g., ubiquitinated histones or if Fun30 is regulated by ubiquitin. However, one cannot presently exclude that CUE motifs and related motifs recognize other ubiquitin related molecules, such as SUMO. Future work will determine the mechanisms by which Fun30 affects gene silencing and if the FUN30/ETL1 putative chromatin remodellers share biological roles.

## Materials and Methods

### Sequence Comparisons

We used PFam (http://www.sanger.ac.uk) [56], and SMART (http://smart.embl-heidelberg.de/) [57] to retrieve the complete collections of putative SNF2 factors for budding yeast *Saccharomyces cerevisiae* and fission yeast *Schizosaccharomyces pombe*. The length and sequences of the domains, including the ATPase domains of each SNF2 factor, were obtained from PFam. In **table 1**, N- and C-ter refer to the sequences of the proteins before and after the SNF2_N domain (see Figure 1 A). Domain assignment for table 1 was as follows: *Saccharomyces cerevisiae* (Sc) Snf2 (1703 aa), SNF2_N domain: from amino acid position 770 to 1065, Helicase-C, 1122-1201; Sc Fun30 (1131 aa), SNF2_N domain: 575-881, Helicase-C, 985-1063; *Schizosaccharomyces pombe* (Sp) Fft1 (944 aa), SNF2_N domain, from 417-699, Helicase-C, 799-877; Sp Fft2 (1284 aa), SNF2_N domain, 553-858, Helicase-C, 959-1037; Sp Fft3 (922 aa), SNF2_N domain, 390-695, Helicase-C, 796-874. Sequence similarity searches were performed using the BLASTp algorithm [58] provided by GeneDB and by SGD, and the PSI-BLASTp algorithm provided by NCBI [59]. Alignments of protein sequences were done using the pairwise alignment algorithms EMBOSS [60], provided by EMBL-EBI (http://www.ebi.ac.uk), and bl2seq [61], provided by NCBI (http://blast.ncbi.nlm.nih.gov). Multiple sequence alignments were generated with ClustalW, available online from EMBL-EBI [62]. For Figure 1B, the alignment generated by ClustalW was adjusted by hand.

### Yeast Culture

Yeast growth and manipulation was according to standard procedures [63]. The strains used are listed in **table 2**. Start- to stop-codon deletion of the Fun30 ORF was by homologous recombination with the kanMX4 cassette using standard procedures [63]. For viability assays, cultures were grown to saturation. The cell density was adjusted to 6×10^7^cells/ ml (OD_600_ = 3) with water, and the cultures were serially diluted by a factor of 10. For each dilution, 3 µl of cell solution were plated on the appropriate agar plate and incubated over several days. Silencing assays were performed as described [32], [33], [35], [37]. For immunodetection, the antibodies used were Anti-V5 mouse monoclonal (Invitrogen) and anti-Histone H3 rabbit polyclonal (Abcam). Microscopy was with an Olympus BX40 microscope, an Olympus objective UplanApo 100x/1.35 oil, an Olympus U-TV 0.5X collector lens and a Soft Imaging System camera MegaViewII. For the budding index experiment, three cultures were analysed and at least 200 cells of each culture were scored. Drugs were tested at following concentrations: thiabendazole: 75–100 mg/ml, brefeldin A: 500 µg/ml, 6-azauracil: 25–300 mg/ ml, etoposide: 200–400 µg/ml, amsacrine: 50–100 µg/ml, zeocin: 25–50 µg/ml, hydroxyurea: 50–300 mM.

**Table 2:** *S. cerevisiae* strains used in this study.

Strain/ Source	Genotype
Y00000 (wt) Euroscarf	*MAT* ***a*** * his3Δl leu2Δ10 met15Δura3Δ0*
Y00389 (*fun30Δ*) Euroscarf	*MAT* ***a*** * his3Δl leu2Δ10 met15Δ ura3Δ0 YAL019w::kanMX4*
JRY7385 Suter et al. 2004 [20]	*MATα his3Δl leu2Δ10 lys2Δ ura3Δ0 can1Δ mfa1Δ::MFA1pr-HIS3*
JRY7385;*fun30Δ* this study	*MATα his3Δl leu2Δ10 lys2Δ ura3Δ0 can1Δ mfa1Δ::MFA1pr-HIS3* YAL019w::kanMX4
JRY7459 Suter et al. 2004 [20]	*MATα his3Δl leu2Δ10 lys2Δ ura3Δ0 can1Δ mfa1Δ::MFA1pr-HIS3 orc5-1::natMX4*
JRY7459;*fun30Δ* this study	*MATα his3Δl leu2Δ10 lys2Δ ura3Δ0 can1Δ mfa1Δ::MFA1pr-HIS3 orc5-1::natMX4 YAL019w::kanMX4*
YLS410 Sussel et al.1993 [32]	*MATα ade2-1 can1-100 his3-11,15 leu2-3,112 trp1-1 ura3-1 HMR::2EDA*
YLS410;*fun30Δ* this study	*MATα ade2-1 can1-100 his3-11,15 leu2-3,112 trp1-1 ura3-1 HMR::2EDA* YAL019w::kanMX4
YLS404 Sussel et al.1993 [32]	*MATα ade2-1 can1-100 his3-11,15 leu2-3,112 trp1-1 ura3-1 GAL^+^ hmrΔA(Δ358-352)::ADE2*
YLS404;*fun30Δ* this study	*MATα ade2-1 can1-100 his3-11,15 leu2-3,112 trp1-1 ura3-1 GAL^+^ hmrΔA(Δ358-352)::ADE*2 *YAL019w::kanMX4*
YLS586 Sussel et al.1993 [32]	*MATα ade2-1 can1-100 his3-11,15 leu2-3,112 trp1-1 ura3-1 GAL^+^ hmrΔB(Δ274-256)::ADE2*
YLS586;*fun30Δ* this study	*MATα ade2-1 can1-100 his3-11,15 leu2-3,112 trp1-1 ura3-1 GAL^+^ hmrΔB(Δ274-256)::ADE2 YAL019w::kanMX4*
YTI312 Iida et al. 2004 [35]	*MAT* ***a*** * bar1Δ::hisG ade2Δ::hisG ura3Δrvs can1-100 his3-11,15 his4 leu2-3 trp1-1 VIIL-adh4::URA3-TEL VR::ADE2-TEL*
YTI312;*fun30Δ* This study	*MAT* ***a*** * bar1Δ::hisG ade2Δ::hisG ura3Δrvs can1-100 his3-11,15 his4 leu2-3 trp1-1 VIIL-adh4::URA3-TEL VR::ADE2-TEL YAL019w::kanMX4*
JS306 (JS311) Smith et al.1999 [34]	*MAT* ***a*** * his3Δ200 leu 2Δ1 met15 Δ0 trp1 Δ63 ura3-167 RDN1::Ty1-MET15 mURA/HIS3* (JS311 is MAT*α*)
JS306;*fun30Δ* This study	*MAT* ***a*** * his3Δ200 leu 2Δ1 met15 Δ0 trp1 Δ63 ura3-167 RDN1::Ty1-MET15 mURA/HIS32 YAL019w::kanMX4*

### Fun30 Expression Plasmids

The coding region of Fun30 excluding the stop codon was amplified by polymerase chain reaction (PCR) from purified genomic DNA and cloned into pYES2.1/V5-His-TOPO (Invitrogen) to create plasmid PFA1. The ATPase-inactive version of Fun30 carries a point mutation in the ATPase domain that replaces lysine at position 603 (AAA) by arginine (AGA) [64]. The point mutation was introduced with the QuickChange XL Site-Directed Mutagenesis kit (Stratagene). The template was PFA1 and the primers were: CGACATGGGTCTAGGTAGAACATGTCAAGTCATTTC and GAAATGACTTGACATGTTCTACCTAGACCCATGTCG. Vector PFA5 contains a version of Fun30 without the CUE motif. From PFA1, a sequence containing the CUE motif was removed by restriction digestion with the enzymes Bsu36I and BclI. The resulting fragment was ligated to a Bsu36I and BclI cleaved DNA fragment amplified from PFA1 using the primers TATATTCCTGAGGATAGGCAGCAA and GGAAGAACATTTCTTGATCACAGA. PFA12 contains a version of Fun30 in which phenylalanine at position 82 (TTC) and proline at position 83 (CCC) have been replaced by alanine (GCC). The template was PFA1 and the primers were GTTAACCTTGCGAGAGAGGCCGCCGATTTCTCTCAAAC and GTTGAGAGAAATCGGCGGCCTCTCTCGCAAGGTTAAC.

### Fluorescence-Activated Cell Sorting (FACS)

A culture was grown to early logarithmic phase and up to 8×10^6^ cells were resuspended in 70% ethanol and incubated at 30°C for 30 min rotating. The cells were washed two times with PBS buffer (150 mM NaCl; 2.7 mM KCl; 16 mM Na_2_HPO_4_; 4 mM KH_2_PO_4_; pH 7.4) resuspended in 500 µl of PI/RNAse staining buffer (BD Biosciences) and incubated at room temperature for 15 min in the dark. The cell suspensions were stored at 4°C and briefly sonicated immediately before use. At least 20000 cells were counted per experiment with a FACSCalibur (BD Biosciences) flow cytometer and the results were processed with the CellQuest software.

### RT-PCR

RNA isolation was with Fast RNA Pro Red Kit (MP Biomedicals) after yeast cells were grown to an OD_600_ of ∼0.7. For RT-PCR, 5 µg RNA was digested with DNAseI, followed by reverse transcription and quantitative PCR.

### Rapid Extraction of Total Protein

Cells of a 5 ml culture grown to saturation were re-suspended in 500 µl of deionized water. 500 µλ of 0.3 M of NaOH were added and the cells were incubated at room temperature for 5 min. After pelleting, they were resuspended in 100 µl of 0.06 M Tris-HCl pH 6.8; 5% (v/v) glycerol; 2% (v/v) SDS; 4% (v/v) β-mercaptoethanol; bromophenol blue and incubated at 95°C for 5 min before immunoblot analysis.

### Chromatin Immunoprecipitation (ChIP)

ChIP was performed essentially as described (http://www.epigenome-noe.net/researchtools/protocol.php?protid=27). Samples were crosslinked for 15 min with 1% formaldehyde. Chromatin samples were sheared to ∼500 bp by sonication. All samples were analyzed by quantitative PCR with a Perkin-Elmer ABI Prism 7700 Sequence Detector System. Oligonucleotides used for the PCR are listed in table 3. Each ChIP was repeated 2–4 times and normalized against input. ChIP was performed using 5 µg of Fun30 antibody or 5 µg of IgG as a control. The rabbit polyclonal anti-Fun30 antibody was generated against a recombinant GST-tagged domain (amino acids 56–136) from Fun30 and affinity purified using a standard protocol [65].

**Table 3:** Oligonucleotides used in this study.

*Primer*	*Sequence (5′-3′)*
a1-F	ttggccttatagagtgtggtcgt
a1-R	aacattgagaacaagagcaagacg
GIT1d-F	gcaccaacaccaatacctacca
GIT1d-R	gccactgctatcttggttattgg
HMR-GIT1-F	tcaattcttgaatctcaacttccatt
HMR-GIT1-R	tccattgatcagtattcatgttcctag
HMR-L probe	ggcgatataatttatcatgttttgg
HMR-R probe	tgtgcaaattccaactaaagga
TelVIR0.5-F	aactgtcggagagttaacaagcg
TelVIR0.5-R	tgaactgtgcatccactcgttag
TelVIR4.8-F	cttgtcttaggcaggctggagt
TelVIR4.8-R	ctgcttcatcatccataaatgatacag
TelVIR9.8-F	ttcatggtaattcgtcgagacagt
TelVIR9.8-R	atccaattgtcaatgagcaggt
TtRNA-F	acactagtaatgtggagatcatcggtt
TtRNA-R	agatgacgatggacgcgaac

### Miccrococcal Nuclease Digestion

Digestions were performed as described [66], with modifications. Yeast cultures were grown in 50 ml YPAD to 0.7 OD_595_, then pelleted. Spheroplasts were generated by digesting pellets with 950 µl 2 mg/ml Zymolyase 100T (MP Biomedicals) for 6 minutes. Spheroplasts were then washed as previously described and digested with 25 or 70 U of micrococcal nuclease for four minutes. The DNA was purified and digested with EcoRV and PciI. 3 µg of digested DNA was separated in a 1.7% TBE-agarose gel and transferred to a nylon membrane. The membrane was analysed using indirect end-labelling with a probe generated by random primer labelling from a 151 bp PCR product prepared using the primers 5′-tgtgcaaattccaactaaagga-3′ and 5′-ggcgataatttatcatgttttcc-3′.
